# Bioinformatics Investigations of Universal Stress Proteins from Mercury-Methylating *Desulfovibrionaceae*

**DOI:** 10.3390/microorganisms9081780

**Published:** 2021-08-21

**Authors:** Raphael D. Isokpehi, Dominique S. McInnis, Antoinette M. Destefano, Gabrielle S. Johnson, Akimio D. Walker, Yessenia A. Hall, Baraka W. Mapp, Matilda O. Johnson, Shaneka S. Simmons

**Affiliations:** 1 College of Science, Engineering and Mathematics, Bethune-Cookman University, Daytona Beach, FL 32114, USA; smithmcinnisd@cookman.edu (D.S.M.); antoinette.m.destefano@students.cookman.edu (A.M.D.); gabrielle.s.johnson@students.cookman.edu (G.S.J.); akimio.d.walker@students.cookman.edu (A.D.W.); yessenia.a.hall@students.cookman.edu (Y.A.H.); williamsbaraka@gmail.com (B.W.M.); 2 College of Nursing and Health Sciences, Bethune-Cookman University, Daytona Beach, FL 32114, USA; johnsonma@cookman.edu; 3 Department of Science and Mathematics, Jarvis Christian College, Hawkins, TX 75765, USA; ssimons@jarvis.edu

**Keywords:** aquatic environments, anaerobic bacteria, bioinformatics, biofilms, *Desulfovibrionaceae*, genomes, mercury, methylation, *Pseudodesulfovibrio mercurii*, stress response, universal stress protein

## Abstract

The presence of methylmercury in aquatic environments and marine food sources is of global concern. The chemical reaction for the addition of a methyl group to inorganic mercury occurs in diverse bacterial taxonomic groups including the Gram-negative, sulfate-reducing *Desulfovibrionaceae* family that inhabit extreme aquatic environments. The availability of whole-genome sequence datasets for members of the *Desulfovibrionaceae* presents opportunities to understand the microbial mechanisms that contribute to methylmercury production in extreme aquatic environments. We have applied bioinformatics resources and developed visual analytics resources to categorize a collection of 719 putative universal stress protein (USP) sequences predicted from 93 genomes of *Desulfovibrionaceae*. We have focused our bioinformatics investigations on protein sequence analytics by developing interactive visualizations to categorize *Desulfovibrionaceae* universal stress proteins by protein domain composition and functionally important amino acids. We identified 651 *Desulfovibrionaceae* universal stress protein sequences, of which 488 sequences had only one USP domain and 163 had two USP domains. The 488 single USP domain sequences were further categorized into 340 sequences with ATP-binding motif and 148 sequences without ATP-binding motif. The 163 double USP domain sequences were categorized into (1) both USP domains with ATP-binding motif (3 sequences); (2) both USP domains without ATP-binding motif (138 sequences); and (3) one USP domain with ATP-binding motif (21 sequences). We developed visual analytics resources to facilitate the investigation of these categories of datasets in the presence or absence of the mercury-methylating gene pair (*hgcAB*). Future research could utilize these functional categories to investigate the participation of universal stress proteins in the bacterial cellular uptake of inorganic mercury and methylmercury production, especially in anaerobic aquatic environments.

## 1. Introduction

Mercury is a trace metal, which in both its organic (methyl mercury) and elemental form (Hg) is known to be highly toxic to all life forms [1,2]. Exposure to mercury can occur through inhalation of toxic elemental mercury vapors [3], through dietary sources and non-dietary sources [4,5]. The presence of methylmercury in aquatic environments and marine food sources is of global concern [2,6]. In the United States, mercury-impaired waterbodies have concentrations of mercury in fish tissue that have exceeded 1.0 mg/kg total mercury [7,8,9,10]. The chemical reaction for the addition of a methyl group to inorganic mercury occurs in diverse bacterial taxonomic groups including the Gram-negative, sulfate-reducing *Desulfovibrionaceae* family of the delta subdivision of proteobacteria [11,12,13]. The genera in the *Desulfovibrionaceae* family include *Bilophila*, *Desulfobaculum*, *Desulfocurvibacter*, *Desulfocurvus*, *Desulfohalovibrio*, *Desulfovibrio*, *Halodesulfovibrio*, *Humidesulfovibrio*, *Lawsonia*, and *Pseudodesulfovibrio* [14]. The availability of whole-genome sequence datasets for some members of the *Desulfovibrionaceae* [15,16,17] presents opportunities to understand the microbial mechanisms that contribute to methylmercury production in water bodies.

The genomes of bacteria that are able to methylate mercury have a two-gene cluster, *hgcA* and *hgcB*, respectively encoding a corrinoid protein and a ferredoxin [12,18]. Bacteria containing the *hgcAB* gene pair occur in a wide range of habitats including extreme natural environments such as coastal dead zones, deep-sea anaerobic sediments, thawing permafrost soils, and hypersaline ecosystems [19]. A list of *Desulfovibrionaceae* genomes predicted to be mercury methylators according to the presence of the *hgcAB* gene pair are available on the data page of the Biogeochemical Transformations at Critical Interfaces project of the Oak Ridge National Laboratory’s Mercury Science Focus Area [12,20]. We are interested in genes that encode the universal stress protein (USP) domain (Protein Family (Pfam) Identifiers: Usp, pfam00582 or PF00582), since they aid bacteria in (1) responding to extreme conditions; and (2) the formation as well as maintenance of adherent bacteria communities termed biofilms [21,22,23,24]. Biofilms can methylate mercury (Hg) at higher rates than unattached bacteria and are a location for mercury methylation in the environment [11]. The USP gene count per genome has not been compiled for the *Desulfovibrionaceae* genomes to enable comparisons between genomes of mercury methylators and those that are not mercury methylators. This research article bridges the knowledge gap on USP gene content of the *Desulfovibrionaceae* genomes.

The universal stress proteins can be composed of one USP domain; two USP domains in tandem; or one or two USP domains together with other functional domains including transporters, kinases, permeases, transferases, and bacterial sensor proteins [23,25]. The three-dimensional structure of universal stress proteins provides evidence for associated molecular functions, biological processes and cellular components. Adenosine-5′-triphosphate (ATP) functions as coenzyme as well as energy molecule [26] and its binding to USPs provides a basis for the functional categorization of USPs [27]. The ATP-binding amino acid motif of G2XG9XG(S/T) categorizes the USP domain into two groups: ATP-binding and non-ATP-binding [27,28]. The categorization of USP domains of the mercury-methylating *Desulfovibrionaceae* will allow for the new bioinformatics investigations and the design of experiments to determine the participation of USPs in bacterial mercury methylation.

Genes for universal stress proteins were predicted from the genome sequencing of *Desulfovibrionaceae* members, including those that methylate mercury and inhabit extreme environments [29,30,31,32]. Thus, the aim of the research reported here was to investigate the protein sequence features encoded by the predicted universal stress protein sequences of mercury-methylating *Desulfovibrionaceae*. We have focused our bioinformatics investigations on protein sequence analytics by developing interactive visualizations to categorize *Desulfovibrionaceae* universal stress proteins by protein domain composition and functionally important amino acids (functional sites).

We applied bioinformatics resources and developed visual analytics resources to categorize a collection of 719 putative universal stress proteins predicted from 93 genomes of *Desulfovibrionaceae*. We identified a subset of 651 *Desulfovibrionaceae* universal stress protein sequences into 488 sequences with one USP domain and 163 with two USP domains. Additionally, the sequences were categorized by (1) the presence of ATP-binding functional sites and (2) the presence of mercury methylation gene pair in the bacterial genome. The findings provide foundations to investigate the participation of universal stress proteins in the bacterial cellular uptake of inorganic mercury and methylmercury production, especially in anaerobic aquatic environments.

## 2. Materials and Methods

### 2.1. Overview—Applying Bioinformatics Resources and Developing Visual Analytics Resources

**Figure 1:**
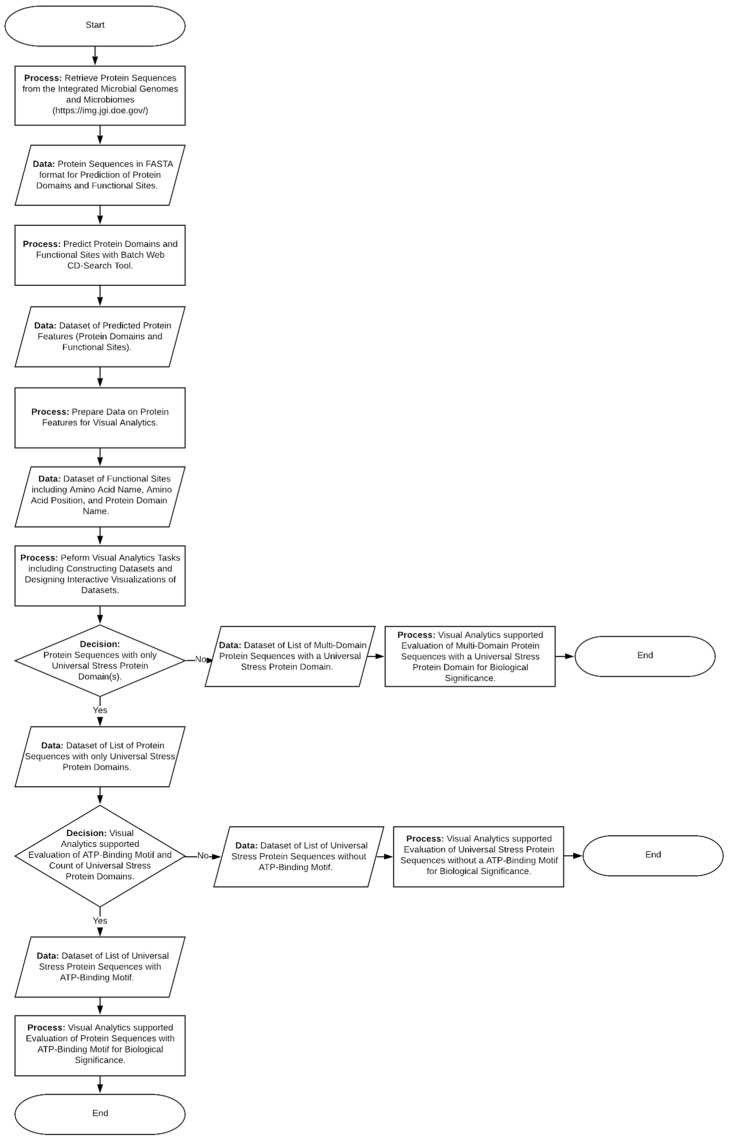
Overview of bioinformatics data investigations of universal stress proteins relevant to bacterial mercury methylation. The process integrates bioinformatics resources and visual analytics resources to categorize universal stress proteins by protein features such as protein domain composition (count and type) as well as the presence of the ATP-binding motif.

The flowchart describing the stages of the bioinformatics investigations is presented in Figure 1. The U.S. Department Joint Genome Institute’s (JGI) Integrated Microbial Genomes and Microbiomes (IMG/M) system [33] was the key bioinformatics resource for collecting and interacting with protein sequence data. We also applied the Batch Web Conserved Domain Search (CD-Search) Tool of the National Center for Biotechnology Information (NCBI) [34] to obtain the number and the protein domain composition as well as the amino acid functional sites.

We typically constructed the results from bioinformatics tasks into datasets that serve as data sources for visual analytics tasks [35]. Bioinformatics tasks that we performed include searching for genes with specific annotation as well as predicting the conserved domains and functional amino acids. The visual analytics tasks include designing interfaces to support interaction, analysis and representation of datasets from bioinformatics tasks [35]. We implemented interactive visualizations (visual representations) in version 2020.4 of Tableau (Tableau, Seattle, WA, USA), a visual analytics software The framework for interaction design for complex cognitive activities with visual representations guided our designs of the interactive visual representations [36,37]. This interaction design framework defines the type of visualizations (e.g., enclosure tables, box plots, and bar plots) and action patterns (e.g., filtering, selecting and transforming) that promotes complex cognitive activities such as decision making, planning, knowledge discovery and understanding [37].

### 2.2. Retrieval of Genome List, Gene List and Protein Sequences annotated with Universal Stress Protein Domain

We applied the Find Genomes and the Find Function tools of the IMG/M system to retrieve, respectively, lists of *Desulfovibrionaceae* genomes and genes annotated with pfam00582. We exported the genome lists and gene lists with annotations from IMG/M into text files for visual analytics tasks. The retrieval of the genome list and gene list in IMG/M generates an Analysis Cart that includes functionalities for exporting protein sequences (in FASTA format). A text file with protein sequences predicted from genes was the input to the Batch Web Conserved Domain Search (CD-Search) Tool of the National Center for Biotechnology Information (NCBI) [34].

### 2.3. Prediction of Protein Domain Composition and Functional Amino Acid Sites

According to the amino acid sequence of the ATP-binding universal stress protein (MJ0577) of *Methanocaldococcus jannaschii*, there are 12 functional sites where amino acids contact the ATP molecule [38]. Thus, we submitted to a bioinformatics resource (NCBI Web Batch CD-Search Tool) a text file containing FASTA formatted amino acid sequences of the *Desulfovibrionaceae* proteins predicted by the IMG/M system to contain the pfam00582 (Usp) domain. We also submitted to the NCBI Web Batch CD-Search Tool a set of 3470 protein sequences predicted from the genome of *Desulfovibrio desulfuricans* ND132. This additional prediction approach could identify potential universal stress proteins that we did not retrieve with the IMG/M pfam00582 function keyword search. It also demonstrates that our categorization process by functional features can be applied beyond the universal stress protein family. The results generated for the protein sequences were Domain hits, Align details, and Features. We downloaded the Features into a file and removed the comment section such that the dataset on functional sites is in a tab-delimited file ready as input for visual analytics.

**Figure A1:**
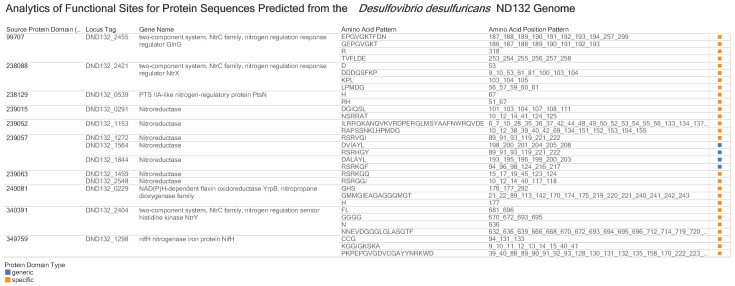
Evidence that the process for analytics of functional sites of protein sequences can be applied beyond the universal stress proteins. The visual representation integrates the protein domain identifier (source protein domain (PSSM-ID)), locus tag, gene name, amino acid pattern and amino acid position pattern. The protein sequences for *Desulfovibrio desulfuricans* ND132 were obtained from the Integrated Microbial Genomes and Microbiomes (IMG/M) system. The protein sequences with annotation for nitrogen metabolism are relevant to our research on the denitrification by microbial communities in oysters [64].

The data fields in the Features file are (1) Query (obtained from FASTA header); (2) Type of protein domain (e.g., specific or superfamily); (3) Title (e.g., Ligand-Binding Site); (4) coordinates (amino acid and position, e.g., P9, V10, D11, C39, M108, G109, R111, G112, G122, S123, V124, T125); (5) complete size (the expected number of functional sites, e.g., 12); (6) mapped size (observed functional sites, e.g., 12); and (7) source domain (protein domain source of functional sites, e.g., 23,812 for the Usp domain). The data file has a record for each protein domain present in the sequence. Thus, it was possible to identify sequences with more than one protein domain including the tandem-type Usp domains. We constructed patterns from the coordinates to facilitate tasks on visual representations, interactions and analyses (such as categorizing and comparing sequences) in a visual analytics software. We developed Perl code to extract patterns from the amino acid coordinates. For example, from coordinates “P9, V10, D11, C39, M108, G109, R111, G112, G122, S123, V124, T125”, the amino acid pattern “PVDCMGRGGSVT” and the amino acid position pattern “9_10_11_39_108_109_111_112_123_124_125” were extracted. Additional information on the Perl code and application beyond the amino acid sequences of the universal stress proteins is presented in the Appendix A (Figure A1). For comparison and accuracy verification of the ATP-binding motif detection procedure, we extracted patterns from the sequences of 10 universal stress proteins from *Mycobacterium tuberculosis* (lab strain H37Rv), whose USPs were extensively investigated for ATP-binding capacity. We performed scripting tasks on computing hardware including a large memory computer cluster (carbonate.uits.iu.edu) configured to support high-performance, data-intensive computing at the National Center Genome Analysis Support (NCGAS), Indiana University [39].

## 3. Results

### 3.1. Count of Universal Stress Protein Genes in Desulfovibrionaceae Genomes

Our search on the Integrated Microbial Genomes and Microbiomes (IMG/M) system for genes encoding the universal stress protein domain (pfam00582) retrieved 716 genes from 93 *Desulfovibrionaceae* genomes. The genera represented in the dataset are *Bilophila*, *Desulfobaculum*, *Desulfocurvibacter*, *Desulfocurvus*, *Desulfohalovibrio*, *Desulfovibrio*, *Halodesulfovibrio*, *Lawsonia*, *Mailhella*, and *Pseudodesulfovibrio*. According to the sequencing status, there were 23 finished genomes, 69 permanent draft genomes and 1 draft genome.

Based on automated Pfam entry annotation, the observed counts of USP gene per genome ranged from 1 to 16. An example genome in each count type is: 1 (*Lawsonia intracellularis* N343); 2 (*Desulfovibrio desulfuricans desulfuricans* ATCC 27774); 3 (*Desulfovibrio fairfieldensis* CCUG 45958); 4 (*Desulfovibrio cuneatus* DSM 11391); 5 (*Desulfovibrio frigidus* DSM 17176); 6 (*Desulfovibrio vexinensis* DSM 17965); 7 (*Desulfovibrio salexigens* DSM 2638); 8 (*Pseudodesulfovibrio piezophilus* C1TLv30); 9 (*Pseudodesulfovibrio aespoeensis* Aspo-2, DSM 10631); 10 (*Desulfovibrio magneticus* RS-1); 11 (*Desulfovibrio gigas* DSM 1382, ATCC 19364); 12 (*Desulfovibrio paquesii* DSM 16681); 13 (*Desulfovibrio vulgaris vulgaris* Hildenborough); 14 (*Halodesulfovibrio marinisediminis* DSM 17456), 15 (*Desulfovibrio alcoholivorans* DSM 5433); and 16 (*Halodesulfovibrio aestuarii aestuarii* ATCC 29578). We observed strains with two genome sequencing projects: *Desulfovibrio alkalitolerans* DSM 16529 (8 USP genes); *Desulfovibrio gigas* DSM 1382, ATCC 19364 (11 USP genes); *Desulfovibrio hydrothermalis* AM13, DSM 14728 (5 USP genes); and *Pseudodesulfovibrio indicus* J2 (7 USP genes).

**Figure 2:**
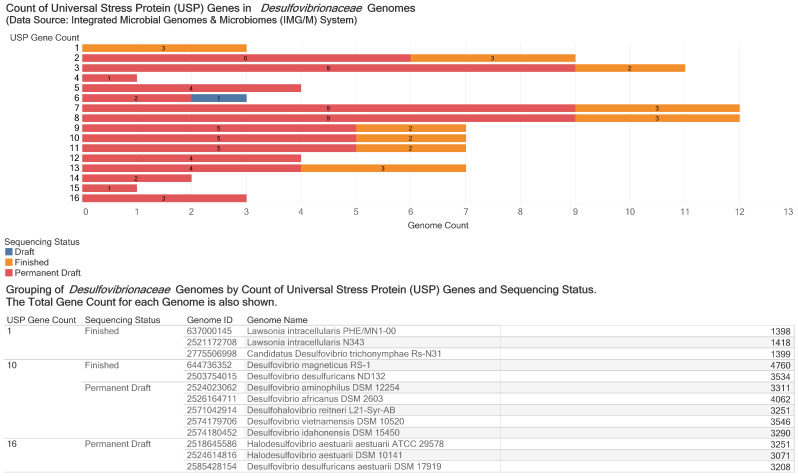
The count of universal stress protein (USP) genes in *Desulfovibrionaceae* Genomes. The bar plot shows the distribution of the gene count by genome count categorized by sequencing status (draft, finished and permanent draft) of the genome. The enclosure table shows examples of genomes with particular gene counts. We have shown genomes with 1, 10 and 16 USP gene count types and the associated total gene count. Among the genomes with USP gene count of 10 is *Desulfovibrio desulfuricans* ND132, a model for bacterial mercury methylation.

Figure 2 is an overview of the distribution of the USP genes in *Desulfovibrionaceae* genomes according to sequencing status and USP gene count. Our bioinformatics investigation for protein domain composition of 3407 protein sequences from *Desulfovibrio desulfuricans* ND132, a bacterial mercury methylation, identified three additional USP genes to make 13 USP genes. Therefore, we collected into a text file 719 FASTA formatted protein sequences annotated to contain the universal stress protein domain.

**Figure 3:**
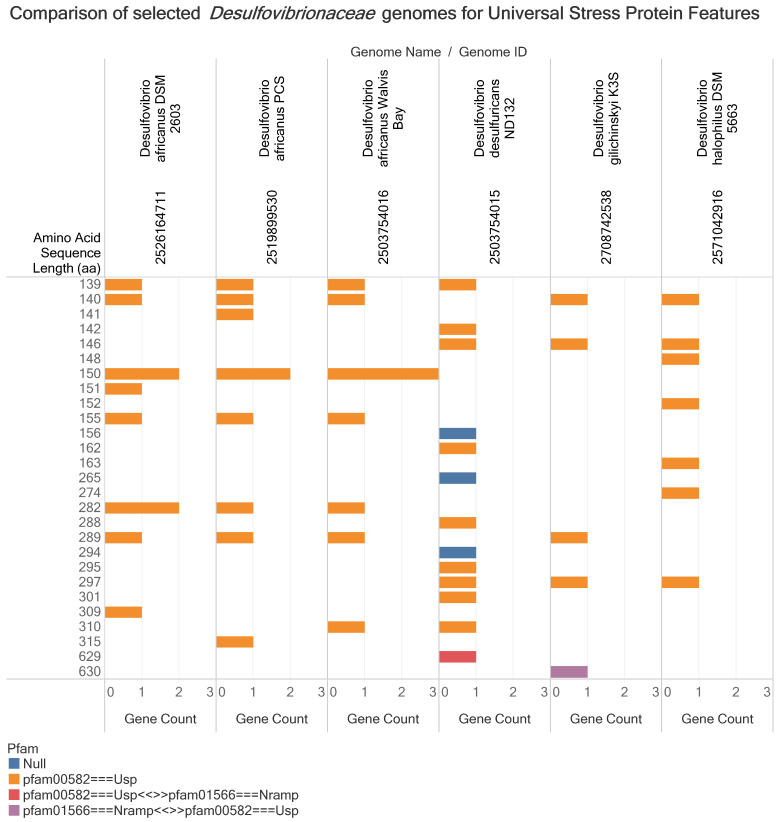
An overview of the protein sequence features for six *Desulfovibrionaceae* genomes including from five that encode the gene pair for mercury methylation. *Desulfovibrio africanus*, *Desulfovibrio desulfuricans* ND132 and *Desulfovibrio halophilus* DSM 5663 are mercury-methylating *Desulfovibrionaceae* species. The comparison visual reveals differences and commonalities in the amino acid sequence length, gene count and protein family (pfam) annotation that were obtained from the annotation in the Integrated Microbial Genomes and Microbiomes (IMG/M) system. For example, among the mercury-methylating *Desulfovibrio africanus* (reclassified as *Desulfocurvibacter africanus*), there is an additional one gene encoding a 150 aa universal stress protein. The color of the bar plots represents the arrangement of the protein domains. Null means no protein domain predicted in the IMG/M resource.

In Figure 3, we present a comparison of the protein sequence features for six *Desulfovibrionaceae* genomes including from five that encode the gene pair for mercury methylation. *Desulfovibrio africanus*, *Desulfovibrio desulfuricans* ND132 and *Desulfovibrio halophilus* DSM 5663 are mercury-methylating *Desulfovibrionaceae* species. Among the genomes of the mercury-methylating *Desulfovibrio africanus* (reclassified as *Desulfocurvibacter africanus*), there is an additional gene for strain Walvis Bay encoding a 150 aa universal stress protein (Figure 3). Furthermore, strain DSM 2603 has an additional 282 aa universal stress protein. In the case of *Desulfovibrio desulfuricans* ND132, the groups of amino acid lengths (aa) observed are 139, 146, 148, 156, 162, 265, 288, 294, 295, 297, 310 and 629. The *Desulfovibrio gilichinskyi* K3S genome encodes five universal stress proteins including a USP with 630 aa. *Desulfovibrio halopilus* DSM 5663 encodes seven universal stress proteins including two protein sequences with lengths 146 aa and 297 aa) that were also predicted from the genomes of *Desulfovibrio desulfuricans* ND132 and *Desulfovibrio gilichinskyi* K3S.

The website to the interactive version of figures generated from visual analytics software is available in the Supplementary Materials section.

### 3.2. Protein Domain Composition and Functional Sites of Desulfovibrionaceae Universal Stress Proteins

The results of the NCBI Batch Web Conserved Domain Search (CD-Search) bioinformatics tool for the 719 protein sequences included predictions on the type and position of the functionally relevant amino acid residues as well as the protein domain(s). The four types of protein domains with functional sites were (1) Universal Stress Protein family; (2) USP domain located between the N-terminal sensor domain and C-terminal catalytic domain of Osmosensitive K+ channel histidine kinase family; (3) old yellow enzyme (OYE)-like Flavin Mono-Nucleotide (FMN)-binding domain; and (4) histidine kinase-like ATPase domain. The NCBI CD-Search Tool identified conserved domains in 718 of the 719 sequences submitted.

We identified 651 universal stress protein sequences that have at least one conserved USP domain model (Position Specific Scoring Matrix Identifier (PSSM-ID) for the USP domain is 23,812). Additionally, we observed 353 patterns (signatures) of amino acid residues (functional sites) associated with 247 amino acid position patterns. For example, an amino acid pattern “AVDVMGHGGSVA” had the highest occurrence in 54 and is associated with amino acid position patterns:

(1) 11_12_13_41_113_114_116_117_127_128_129_130 (7 sequences)
(2) 10_11_12_40_112_113_115_116_126_127_128_129 (44 sequences)
(3) 9_10_11_39_111_112_114_115_125_126_127_128 (3 sequences).

**Figure 4:**
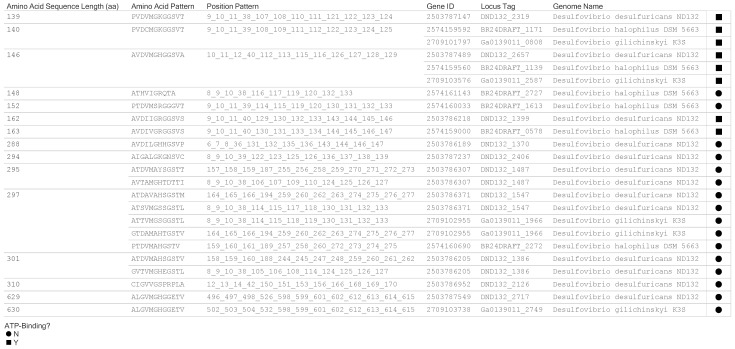
The patterns of amino acid type and amino acid positions for selected *Desulfovibrionaceae* universal stress proteins. We annotated the proteins for the presence of ATP-binding motif (filled shape, square for presence of ATP-binding motif and circle for absence of ATP-binding motif). *Desulfovibrio desulfuricans* ND132 and *Desulfovibrio halophilus* DSM 5663 are mercury methylating. *Desulfovibrio gilichinskyi* K3S is included for comparison of the 629 aa universal stress protein from *Desulfovibrio desulfuricans* ND132.

**Figure 5:**
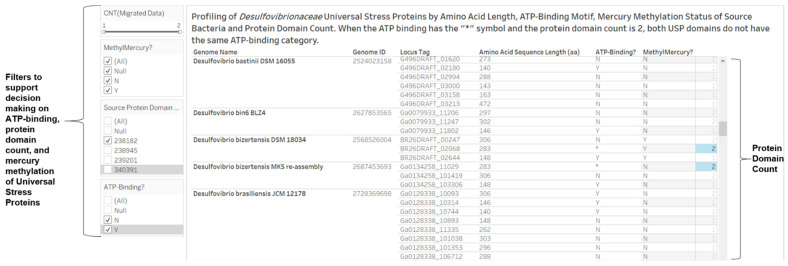
Profiling of *Desulfovibrionaceae* universal stress proteins by amino acid length, ATP-binding motif, mercury methylation status of source bacteria and protein domain count. When the ATP-binding has the “*” symbol and the protein domain count is 2, both USP domains do not have the same ATP-binding category (BR26DRAFT_02068 and Ga0134258, which are 283 aa USP of strains of *Desulfovibrio bizertensis*). Protein domain count of “1” indicates protein sequence has only one USP domain.

The amino acid position pattern of 9_10_11_39_111_112_114_115_125_126_127_128 was restricted to sequences from three *Bilophila* species with Locus Tags (HMPREF0178_03304, T370DRAFT_02139, and HMPREF0179_03080). Based on the ATP-binding motif of G2XG9XG(S/T), our algorithm (a calculated field in the visual analytics software) classified the 353 functional site amino acid patterns into two motif types: 236 (non-ATP-binding motif) and 117 (ATP-binding motif). We designed a visual representation to grouped the 651 protein sequences by amino acid sequence length, amino acid pattern and amino acid position pattern (Figure 4 shows a subset for three genomes: *D. desulfuricans* ND132, *D. halophilus* DSM 5663 and *D. gilichiniskyi* K3S). The design allowed us to identify proteins with identical amino acid sequence length and pattern of functional site (for example, the 146 aa and 297 aa sets encoded by the three genomes). We observed 13 types of functional site patterns from 10 of the 13 universal stress protein sequences predicted from *Desulfovibrio desulfuricans* ND132 (Figure 5). Protein sequences for DND132_1399, DND132_2319, and DND132_2657 have evidence for ATP-binding. The NCBI CD-Search did not report functional sites for DND132_1176 (IMG/M Gene ID 2503785994), DND132_1371 (IMG/M Gene ID 2503786190), and DND132_1376 (IMG/M Gene ID 2503786195).

**Figure A2:**
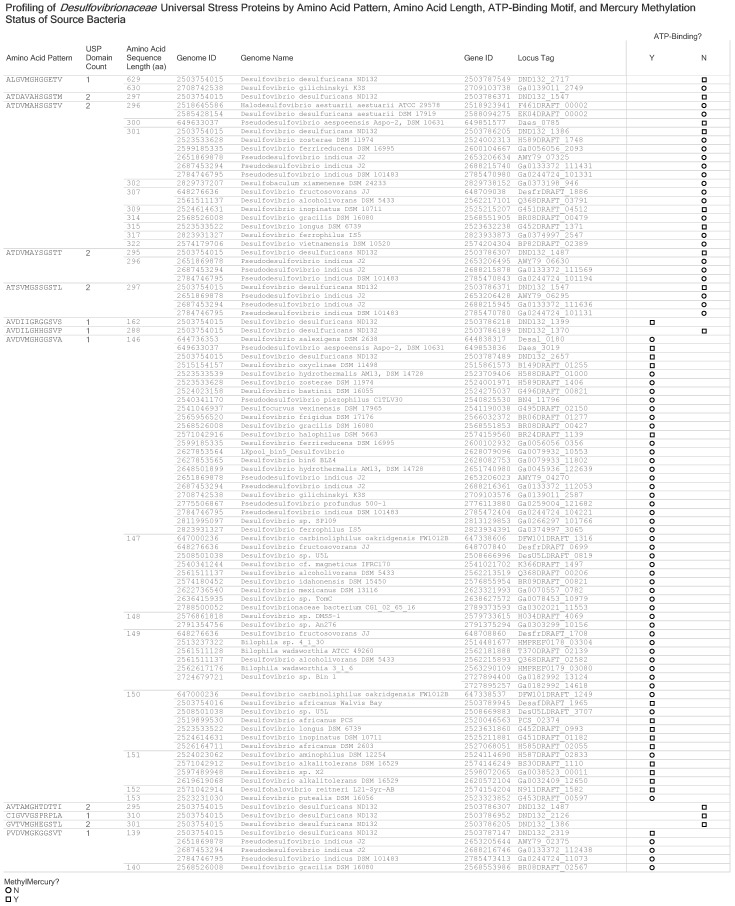
Profiling of 92 *Desulfovibrionaceae* universal stress proteins by amino acid pattern (functional sites for ATP binding), protein domain count, amino acid length, ATP-binding motif, and mercury methylation status of source bacteria. The interactive version of the visual is available at https://public.tableau.com/app/profile/qeubic/viz/uspdesulfofamily/figureB1 (accessed on 20 August 2021).

The DND132_2657 gene for a 146 aa protein was among the 54 *Desulfovibrionaceae* USP genes encoding the ATP-binding functional site pattern “AVDVMGHGGSVA”. The protein domain arrangement of DND132_2717, a 629 aa protein sequence, comprised of a metal ion transporter domain and a USP domain with functional sites that do not conform with the ATP-binding motif. Comparison of amino acid sequence length and protein domain composition provided evidence that among the *Desulfovibrionaceae* genomes investigated the combination of metal ion transport domain and universal stress protein unique to *D. desulfuricans* ND132 and *D. gilichinskyi* K3S (previously named *Desulfovibrio algoritolerance* K3S). The IMG/M Gene ID, Locus Tag and amino acid sequence length for the equivalent gene of DND132_2717 in *Desulfovibrio gilichinskyi* K3S is 2709103738 and Ga0139011_2749 and 630 aa, respectively. Both protein sequences have an identical amino acid pattern of “ALGVMGHGGETV”. Figure A2 in the Appendix A presents profiling of 92 *Desulfovibrionaceae* USPs by amino acid pattern, amino acid length, ATP-Binding motif, and mercury methylation status of source bacteria.

**Figure 6:**
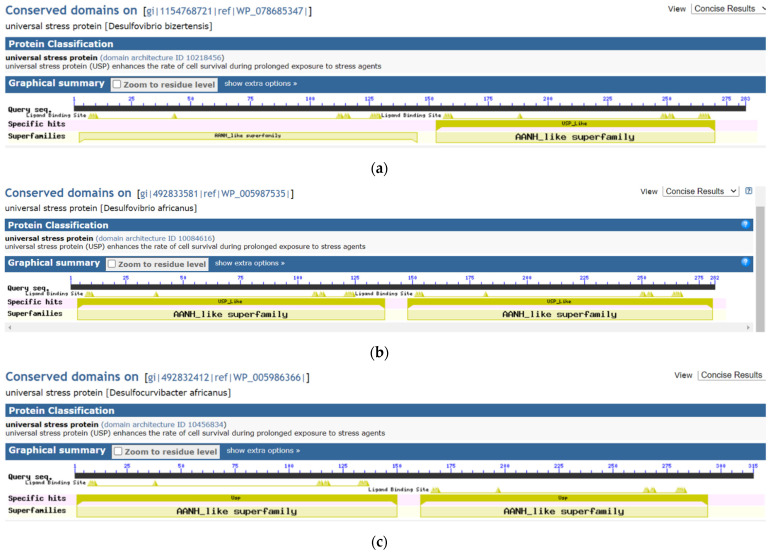
Protein domain compositions of three tandem-type universal stress proteins from mercury-methylating *Desulfovibrionaceae* bacteria. (**a**) In WP_0005987535 of *Desulfovibrio bizertensis* DSM 18034, the USP domains do not have the same ATP-binding category. (**b**) In WP_005986366 of *Desulfocurvicter africanus* PCS, the USP domains are both ATP-binding. (**c**) In WP_005986366 of *Desulfocurvicter africanus* PCS, the two USP domains are not ATP-binding. Interactive version of each protein domain composition is available at the National Center for Biotechnology Information (NCBI) Conserved Domains resource by searching for the protein sequence identifier.

In Figure 5, the visual analytics design integrates the amino acid length, ATP-binding prediction of the USP domain, and the mercury methylation status of the source bacteria. The view among other functions allow for the categorization of a tandem-type USP by the types of ATP-binding prediction (Y = ATP-binding for both domains; N = Both domains are not ATP-binding; and * = One domain is ATP-binding and other does not bind ATP). The findings were confirmed with the NCBI Conserved Domains resource for three tandem-type USPs encoded in *Desulfovibrionaceae* genomes that encode the hgcA and hgcB proteins (Figure 6).

## 4. Discussion

We have conducted bioinformatics investigations on the universal stress proteins encoded in *Desulfovibrionaceae* genomes including genomes of strains that methylate mercury. Prior to our study, the characterization of *Desulfovibrionaceae* USPs was limited to genome-wide transcriptome or proteome analyses [17,32,40]. Our report provides findings from protein sequence analytics to guide further research on the molecular functions, biological processes and cellular components associated with *Desulfovibrionaceae* universal stress proteins (USPs). In our prior publications, we have applied bioinformatics and developed visual analytics resources to understand the universal stress proteins of taxonomic groups namely viridiplantae, *Bacillus*, *Schistosoma*, *Alcanivorax*, *Brucella* and *Lactobacillus* [35,41,42,43,44,45,46]. In this report, we have made noteworthy findings on a collection of 719 *Desulfovibrionaceae* USPs regarding (1) protein domain arrangement; and (2) functional amino acid residues (Figure 1).

The observed counts of USP gene per genome among 93 *Desulfovibrionaceae* genomes ranged from 1 to 16 (Figure 2). The count of USP genes per genome could reflect the diverse phenotypic properties and habitats of the *Desulfovibrionaceae* members. The number of USP gene per genomes of *Escherichia coli* and *Mycobacterium tuberculosis* are six and ten, respectively [23,47]. The genomes of three *Halodesulfovibrio aestuarii* strains had 16 USP genes, the highest observed among the 93 genomes investigated. The *Halodesulfovibrio* species tolerates up to 6% (*w*/*v*) sodium chloride (NaCl) with optimum growth at 1.5–3.5% (*w*/*v*) [48]. Future research could investigate the relationship between universal stress protein function and mercury methylation in the NaCl tolerant *Desulfovibrio halophilus* DSM 5663.

The genomes of three *Desulfovibrio desulfuricans desulfuricans* strains namely ATCC 27774, DSM 642 and DSM 7057 had only two USPs compared to 10 USP genes (retrieved from the IMG/M resource) for strain *D. desulfuricans* ND132. The finding of an excess number of USP genes further supports the reclassification of strain ND132. Recent phylogenetic analyses have clustered strain ND132 with validly published and reclassified members of *Pseudodesulfovibrio* genus including mercury-methylating *Pseudodesulfovibrio hydrargyri* BerOc1 [49,50]. A February 2021 publication formally described strain ND132 as *Pseudodesulfovibrio mercurii* ND132 [51]. We recommend comparative analysis of the universal stress proteins of *Pseudodesulfovibrio* strains to determine the effects of protein domain composition and genomic context of USP genes on stress response and methylmercury production.

Based on the ATP-binding motif of G2XG9XG(S/T), our algorithm (a calculated field in the visual analytics software, Tableau) categorized the 353 functional site amino acid patterns into two motif types 236 (non-ATP-binding motif) and 117 (ATP-binding motif) (Figure 5). For tandem-type USPs, we developed visual analytics views that provides three categories according to ATP-binding (Figure 6). Future research can investigate the biological significance of these categories. The *Desulfovibrio desulfuricans* ND132 protein sequences for DND132_1399, DND132_2319, and DND132_2657 have evidence for ATP-binding. Research investigations are required to understand the molecular function, biological processes and cellular components of the predicted ATP-binding USPs of strain ND132. The ATP-binding universal stress proteins are predicted to function in energy-dependent biological processes [52]. Examples of ATP (energy)-regulated processes are: (1) the regulation of entry into chronic persistent growth phase in *Mycobacterium tuberculosis* [28]; (2) the response to acid stress condition during the exponential growth phase in *Listeria innocua* [53]; (3) susceptibility of *Mycobacterium tuberculosis;* and (4) survival of *Mycobacterium smegmatis* in human monocyte cells [52]. The visual analytics resource accompanying this report provides a resource for interacting with the datasets on predicted ATP-binding status. Further, the genomic context or neighborhood of the USP genes can provide insights on the molecular function, biological processes and cellular components of the universal stress proteins of strain ND132.

Among the four universal stress protein sequences of strain ND132 that contain two protein domains (DND132_1487, DND132_1547, DND132_1386, and DND132_2717), only DND132_2717 (a 629 aa protein) has a metal ion transporter domain (pfam01566 or PF01566: natural resistance-associated macrophage protein (NRAMP) domain) (Figure 3). The transmembrane NRAMP family of transporters function as divalent metal ion transporters from bacteria to humans [54]. Thus, we recommend research to determine (1) if DND132_2717 transports inorganic divalent mercury ions (Hg^2+^); (2) if DND132_2717 localizes to the membrane; and (3) if DND132_2717 function is regulated by the universal stress protein domain. The divalent metal cation transporter is listed among metal transporters impacted by the deletion of *hgcAB* genes of strain DND132 [55]. A yeast divalent cation transporter DMT1 of participates in the uptake of inorganic mercury [56]. Research publications on the uptake of inorganic mercury in mercury-methylating *Desulfovibrionaceae* species and related organisms could guide these future studies [57,58,59,60].

The results of bioinformatics investigations are influenced by several factors including the version of software and updates to datasets. The taxonomy of the *Desulfovibrionaceae* has recently been updated including reclassification and formal description of strain ND132 [51,61,62]. Our investigation has considered these limitations and have included information on when the investigations were conducted. We also use multiple approaches, databases and genomic data to achieve consensus results. We have provide results as part of visual analytics resources to support the formulation of new problems for investigations beyond those reported here. The visual analytics resources can also serve as resources for educational interventions for learning biological data investigation [63]. We are also using the methods and findings to investigate denitrification potential of bacterial communities of Eastern Oyster (*Crassostrea virginica*) found in benthic environments [64].

## 5. Conclusions

We have determined protein domain composition and ATP-binding functional sites to categorize a collection of 719 genes predicted to encode the universal stress protein (USP) domains in 93 *Desulfovibrionaceae* genomes. The key findings are the categories of universal stress protein sequences according to (1) USP domain count; and (2) presence of ATP-binding motif (functional sites). We have identified 651 *Desulfovibrionaceae* universal stress protein sequences, of which 488 sequences had only one USP domain and 163 had two protein USP domains. The 488 single USP domain sequences were further categorized into 340 sequences with ATP-binding motif and 148 sequences without ATP-binding motif. The 163 double USP domain sequences were categorized into (1) both USP domains with ATP-binding motif (3 sequences); (2) both USP domains without ATP-binding motif (138 sequences); and (3) one USP domain with ATP-binding motif (21 sequences). We developed visual analytics resources to facilitate the investigation of these categories of datasets in the presence or absence of the mercury-methylating gene pair (hgcAB). Future research could utilize these functional categories to investigate the participation of universal stress proteins in the bacterial cellular uptake of inorganic mercury and methylmercury production, especially in anaerobic aquatic environments.

## Data Availability

The Perl code, input sequences, and output datasets used in this report for the analytics of the conserved protein domains are available on the GitHub software development platform at https://github.com/qeubic/protein_features (accessed on 20 August 2021).

## Supplementary Materials

The online versions of the interactive analytics resources produced are available at https://public.tableau.com/app/profile/qeubic/viz/uspdesulfofamily/overview. Figure A1: Evidence that the process for analytics of functional sites of protein sequences can be applied beyond the universal stress proteins; Figure A2: Profiling of 92 *Desulfovibrionaceae* universal stress proteins by amino acid pattern (functional sites for ATP binding), protein domain count, amino acid length, ATP-binding motif, and mercury methylation status of source bacteria.

## Appendix A

The computer programming language code for preparing the output from NCBI Conserved Domain search can be applied to any collection of fasta-formatted protein sequences or list of NCBI protein identifiers. In this report, we applied the predicted protein sequences from the genome sequence of *Desulfovibrio desulfuricans* ND132. The input sequences are from the Integrated Microbial Genomes/Microbiomes (IMG/M) resource. The Perl code, input sequences, and output datasets used in this report for the analytics of the conserved protein domains are available on the GitHub software development platform at https://github.com/qeubic/protein_features (accessed on 20 August 2021).

A visual analytics resource for protein sequence analytics is available at https://public.tableau.com/app/profile/qeubic/viz/uspdesulfofamily/figureA1 (accessed on 20 August 2021). The designs are for interacting with the data on the protein families including protein domain composition and functional sites. Figure A1 shows that the protein sequence analytics procedure can be applied to other protein groups (e.g., nitrogen metabolism protein groups that have “nitr” in the gene/protein name).

The integration of disparate features of the universal stress proteins from 50 *Desulfovibrionaceae* genomes can facilitate comparative analysis and planning of future research. Therefore, we have constructed a visualization that integrates the amino acid pattern, amino acid length, ATP-binding motif, and mercury methylation status of source bacteria (Figure A2. The mercury methylation status was obtained from the data page of the Biogeochemical Transformations at Critical Interfaces project of the Oak Ridge National Laboratory’s Mercury Science Focus Area [12,20]. A total of 92 *Desulfovibrionaceae* USPs were profiled according to 12 amino acid patterns, 28 amino acid lengths, two ATP-binding motifs, and mercury methylation status of the source bacteria.

The 92 USPs included 60 USPs with ATP-binding motifs, 27 USPs from 12 genomes of mercury methylators and 15 single-domain USPs that are ATP-binding and from the genomes of mercury methylators. The genomes encoding mercury methylation are *Desulfohalovibrio reitneri* L21-Syr-AB, *Desulfovibrio africanus* DSM 2603, *Desulfovibrio africanus* PCS, *Desulfovibrio africanus* Walvis Bay, *Desulfovibrio alkalitolerans* DSM 16529, *Desulfovibrio desulfuricans* ND132, *Desulfovibrio halophilus* DSM 5663, *Desulfovibrio inopinatus* DSM 10711, *Desulfovibrio longus* DSM 6739, *Desulfovibrio oxyclinae* DSM 11498, *Desulfovibrio* sp. X2, and *Pseudodesulfovibrio aespoeensis* Aspo-2, DSM 10631 [18].
